# Involvement of Bradykinin B2 Receptor in Pathological Vascularization in Oxygen-Induced Retinopathy in Mice and Rabbit Cornea

**DOI:** 10.3390/ijms19020330

**Published:** 2018-01-23

**Authors:** Erika Terzuoli, Lucia Morbidelli, Ginevra Nannelli, Antonio Giachetti, Sandra Donnini, Marina Ziche

**Affiliations:** Department of Life Sciences, University of Siena, Via A. Moro 2, 53100 Siena, Italy; terzuoli8@unisi.it (E.T.); lucia.morbidelli@unisi.it (L.M.); nannelli5@student.unisi.it (G.N.); okkamm@gmail.com (A.G.)

**Keywords:** bradykinin, B2R antagonist, retinal endothelial cells, angiogenic factors, oxygen-induced retinopathy

## Abstract

The identification of components of the kallikrein–kinin system in the vitreous from patients with microvascular retinal diseases suggests that bradykinin (BK) signaling may contribute to pathogenesis of retinal vascular complications. BK receptor 2 (B2R) signaling has been implicated in both pro-inflammatory and pro-angiogenic effects promoted by BK. Here, we investigated the role of BK/B2R signaling in the retinal neovascularization in the oxygen-induced retinopathy (OIR) model. Blockade of B2R signaling by the antagonist fasitibant delayed retinal vascularization in mouse pups, indicating that the retinal endothelium is a target of the BK/B2R system. In the rabbit cornea assay, a model of pathological neoangiogenesis, the B2 agonist kallidin induced vessel sprouting and promoted cornea opacity, a sign of edema and tissue inflammation. In agreement with these results, in the OIR model, a blockade of B2R signaling significantly reduced retinal neovascularization, as determined by the area of retinal tufts, and, in the retinal vessel, it also reduced vascular endothelial growth factor and fibroblast growth factor-2 expression. All together, these findings show that B2R blockade reduces retinal neovascularization and inhibits the expression of proangiogenic and pro-inflammatory cytokines, suggesting that targeting B2R signaling may be an effective strategy for treating ischemic retinopathy.

## 1. Introduction

The kallikrein–kinin system (KKS) has long been recognized as a key player of inflammatory processes in various organs [1]. In particular, components of the KKS, including plasma kallikrein, factor XII and high-molecular-weight kininogen (HK), are increased in the vitreous of patients with diabetic retinopathy and have been associated with retinal vascular inflammation and neoangiogenesis [2]. Bradykinin (BK), generated by the plasma kallikrein proteolytic activity on HK, primarily mediates KKS actions.

BK exerts potent pro-inflammatory and pro-angiogenic effects through the activation of two G-protein-coupled receptors, BK receptor 1 and 2 (B1R, B2R), widely expressed in vascular tissues, including retinal vessels [3]. Previously, we demonstrated that, at nanomolar concentration, BK promotes angiogenesis in rabbit corneas [4], while, at higher concentrations, it enhances vascular permeability and promotes an inflammatory-related angiogenesis in in vitro and in vivo models, events, which are significantly inhibited by the B2R antagonist, fasitibant [5]. BK/B2R signaling was also reported to induce leakage in post-capillary venules of rat mesentery, and angioedema in both C1-INH null mice and in humans [6,7].

In endothelial cells, we demonstrated that BK/B2R signaling promotes cell proliferation and migration through activation of the pro-inflammatory nuclear factor kB and by the upregulation of cyclooxygenase-2, prostaglandin-E2 and vascular endothelial growth factor (VEGF) [5]. A similar pro-inflammatory and proangiogenic pattern has also seen upregulated in rat retinal microvessel dysfunction [8].

Other studies suggested that the BK/B2R-dependent angiogenic response might be mediated by the upregulation of fibroblast growth factor-2 (FGF-2) and through transactivation of the VEGF-receptor 2 signaling [4,9]. VEGF is the major proangiogenic and pro-inflammatory factor released in the retina under ischemic and inflammatory conditions [8,10]. FGF-2, in turn, mediates survival and sprouting of endothelial cells under hypoxic conditions and appears to be required for the VEGF effects [11,12,13,14]. FGF-2 stimulates VEGF secretion from vascular smooth muscle, endothelial and Müller cells and synergizes with VEGF in promoting the proliferation of retinal microvascular endothelial cells and pericytes [15,16]. Furthermore, FGF-2 and VEGF levels, as the KKS components, are significantly increased in vitreous from experimental models of retinal diseases [17], as well as at sites of chronic inflammation [18], where both growth factors and BK appear to be the key metabolic mediators of inflammatory responses, including vasoactive responses [19,20].

Here, we investigate the role of BK/B2R signaling in two in vivo models of neovascularization, the rabbit cornea assay and the mouse retinal vascularization assay. We demonstrate that the B2R signaling is involved in physiological retinal vascularization. In fact, the B2R blockade by a selective antagonist significantly delayed the extension of retinal vessels in mouse pups. At high concentration, BK significantly induced pathological vessel sprouting in avascular rabbit cornea accompanied by an inflammatory response. Kallidin, a B2R agonist, mimicked the activity of BK in rabbit cornea, inducing a marked neoangiogenesis associated with a persistent corneal opacity indicating that B2R signaling plays a key role in inflammation-related angiogenesis. In the OIR model, characterized by hypoxia-induced pathological pre-retinal neovascularization, the B2R blockade reduced retinal neovessels, as determined by the area of retinal tufts (unorganized, small-caliber vessels, also termed pathological neovessels), and the expression of VEGF and FGF-2 in pathological retinal vessels, suggesting that drugs targeting B2R signaling might have a role in proliferative retinal diseases.

## 2. Results

### 2.1. B2R Blockade Reduces the Extent of Retinal Vascularization and Maturation in Mouse Pups

**Figure 1:**
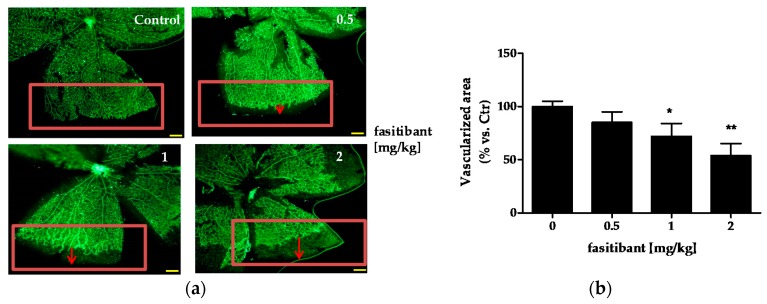
Fasitibant reduces the extent of retinal vascularization in mouse pups. (**a**) pups were treated with or without fasitibant (NaCl 0.9% = Control; fasitibant, 0.5 to 2 mg/kg, in 50 µL, i.p.) and then retinas were dissected at P8 and stained with isolectin, IB4-488. Magnitude 4×, scale bar = 100 µm; (**b**) quantification of retinal vessels. Retinal whole-mounts from P8 pups were stained for endothelial cells with IB4-488. For measurement of the vascularized area at the retinal edges, the retinal edges were outlined with image-processing software (Photoshop Adobe Systems, Adobe Photoshop Elements 11). The density of vascularization in the outlined areas was quantified by ImageJ software, version 2.0.0-rc-43/1.50e, U.S. National Institutes of Health, Bethesda, MD, USA; and expressed in relation to the control areas (% vs. Ctr). Data are the mean of 10 outlined areas, obtained from at least five flower-like structures. Arrows indicate the distance from vascularized area to retinal edge. * *p* < 0.05, ** *p* < 0.01 vs. Ctr.

Mouse pups have an immature retinal vascularization at birth. The superficial vascular plexus forms during the first week after birth by radial outgrowth of vessels from the optic nerve into the periphery, reaching the retinal edges at approximately postnatal day (P) 8. Preliminary experiments were performed on mice (C57Bl/6) from P4 to P8 to investigate the B2R signaling activity on physiological radial outgrowth of retinal vessels and the maximum tolerate dose of the B2R antagonist fasitibant. Mice were treated daily with fasitibant (0.5, 1 and 2 mg/kg) and then retinal vascularization was evaluated by immunohistochemistry. In all mice, no effects were observed after vehicle treatment (daily intraperitoneal, i.p., administration NaCl 0.9% treatment in 50 μL, Control, Figure 1a,b). Fasitibant (daily i.p. treatment in 50 μL, from P4 to P7), significantly delayed the extent of retinal vascularization in a dose dependent manner, evaluated at P8, maximal activity being at 2 mg/kg (Figure 1a,b), without signs of toxicity. At higher doses, fasitibant showed toxicity after 1–2 treatments, estimated as reduced mice motility, failure to eat, and respiratory distress.

**Figure 2:**
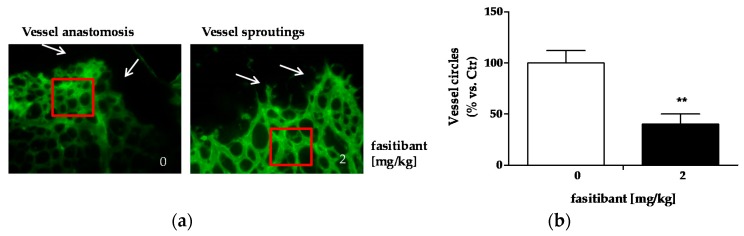
Fasitibant delays retinal vessel maturation in mouse pups. (**a**) Retinas dissected from pups at P8, treated with or without fasitibant (2 mg/kg) were stained for IB4-488. Arrows indicate vessel anastomosis in left panel (Control condition) and vessel sprouting (right panel, fasitibant treatment). Magnitude 40×; (**b**) quantification of vessel density measured as number of vessel circles. For measurement of vascular density, a square close to the retinal edge was performed with Photoshop (Adobe Systems) and the density of vascularization in the square was quantified by ImageJ software, and expressed in relation to the control areas (% vs. Ctr). Data are the mean of 10 squares, obtained from at least five flower-like structures. ** *p* < 0.01 vs. Ctr.

At 2 mg/kg, fasitibant perturbed the retinal vascular pattern, delaying vessel fusion, indicative of vessel maturation, and reducing vessel density (Figure 2a,b).

### 2.2. B2R Signaling Promotes Pathological Neoangiogenesis

**Figure 3:**
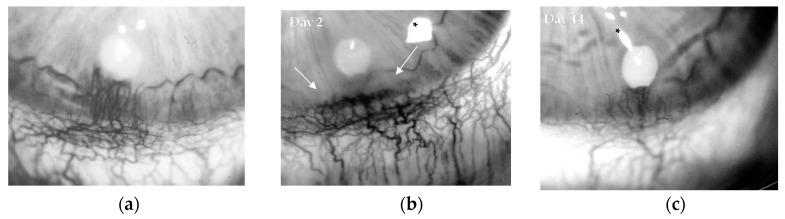
Bradykinin (BK) stimulates angiogenesis through the bradykinin 2 receptor (B2R). Representative images of rabbit corneas implanted with: (**a**) BK (1 µg/pellet) (panel a, day 14) and (**b**) kallidin (5 µg/pellet) at day 2 or (**c**) day 14. Asterisks mark flash artifacts. White arrows indicate corneal opacity with loss of corneal clarity and transparency. Original magnification 18×.

In order to investigate whether B2R signaling was also involved in pathological neoangiogenesis in adult tissues, inflammatory concentrations of BK or kallidin were evaluated in the avascular rabbit cornea assay. This in vivo model was used to specifically investigate whether B2R signaling was associated to pathological neoangiogenesis, thus BK and the selective B2 receptor agonists, kallidin, were implanted in the corneal stroma as slow-release preparations. BK (1 µg/pellet) induced a marked pathological neoangiogenesis (Figure 3a), characterized by tortuous vascular sproutings, which were associated with mild corneal opacity, a sign of increased vessel permeability and inflammatory response. Similarly, the selective B2 receptor agonist kallidin at 5 μg/pellet induced significantly corneal angiogenesis (Figure 3b,c), associated with loss of corneal transparency (Table 1), while a modest effect was observed with kallidin at 1 μg/pellet. As BK and kallidin were nearly equipotent (EC50 10**^−^**^12^ M) [21], the data indicates that BK effect on pathological neoangiogenesis was mainly mediated by B2R signal activation. 

### 2.3. Fasitibant Reduces Retinal Neovascularization in the OIR Model

**Figure 4:**
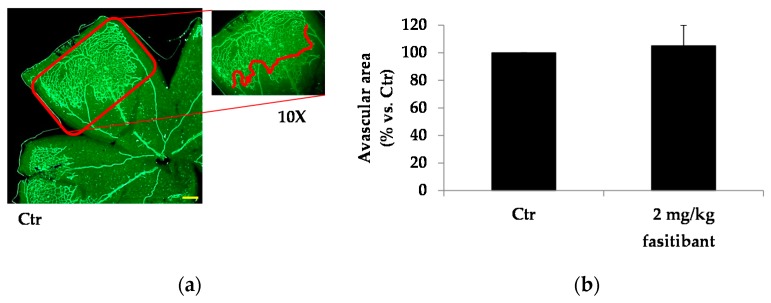

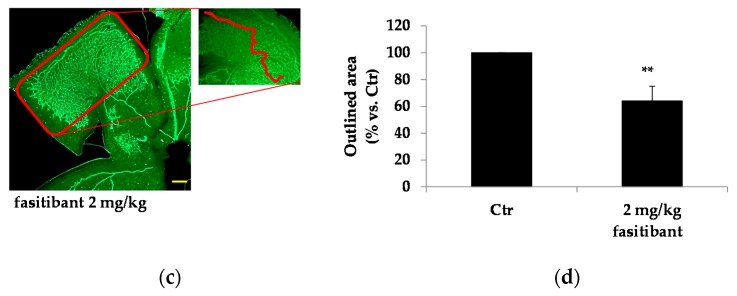
B2R signaling inhibition reduces pathological vessels in Oxygen-induced retinopathy (OIR) model. Representative OIR flat mounted retinas stained for IB4-488: (**a**) vehicle treated and (**c**) fasitibant treated. Left panels: magnification 4×, scale bar = 100 µm; Right panels: magnification 40×; (**b**) quantification of avascular area and (**d**) area with neovascular tufts. The extent of neovascular tufts area was quantitatively evaluated outlining with Photoshop the border of tufts between the vascularized peripheral area and the avascular central area and the density of neovascular tufts area was quantified by ImageJ software. Each column represents the mean ± SD of data from 10 different quadrants derived from 10 retinas. ** *p* < 0.01 vs. Ctr.

Next, based on above results, we assessed whether B2R signaling contributed to the pathogenesis of retinal microvascular complications by using the model of oxygen-induced retinopathy (OIR) in mice, which is well characterized by retinal vascular regression and hypoxia-induced pathological pre-retinal neovascularization. In OIR mice, a large avascular area was observed at the center of the retina, while the mid-peripheral area showed regrowth of superficial vessels leading to pre-retinal neovascular tufts, which were, mostly, at the border between the vascularized peripheral area and the avascular central area. Fasitibant did not affect the extension of the avascular central area (Figure 4a–d), while significantly reducing the vessel tufts area (Figure 4a–d).

**Figure 5:**
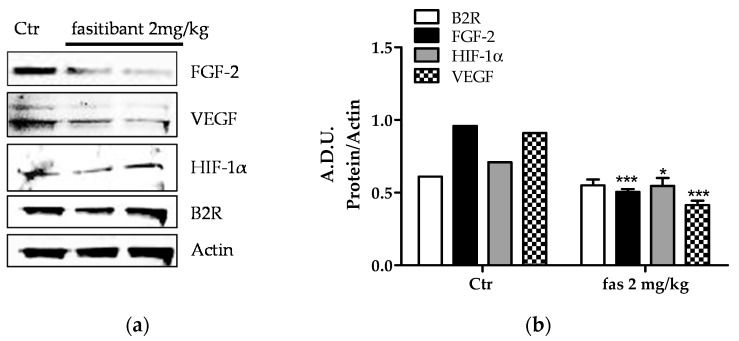
BK/B2R signaling inhibits proangiogenic growth factor expression in OIR mice. (**a**) representative blots of fibroblast growth factor (FGF-2), vascular endothelial growth factor (VEGF), hypoxia inducible factor (HIF(-1α and B2R expression in OIR mice treated with or without fasitibant (fas) given at 2 mg/kg. Two groups of fasitibant 2 mg/kg are reported in which each lane is representative of four pooled retinas (**b)** lane quantification with ImageJ. Data are reported as arbitrary density unit (A.D.U.). FGF-2, VEGF, HIF-1α and B2R lanes were normalized to β-actin. Fas bands are the mean of the two representative groups (8 retinas) reported in panel (**a**). * *p <* 0.05; *** *p* < 0.001 vs. Ctr.

Furthermore, in OIR mice, B2R blockade markedly reduced FGF-2 and VEGF protein expression in retinal microvessels (Figure 5a). It also reduced, at a lesser extent, hypoxia inducible factor (HIF)-1α expression, without affecting B2R levels (Figure 5a,b). 

## 3. Discussion

**Figure 6:**
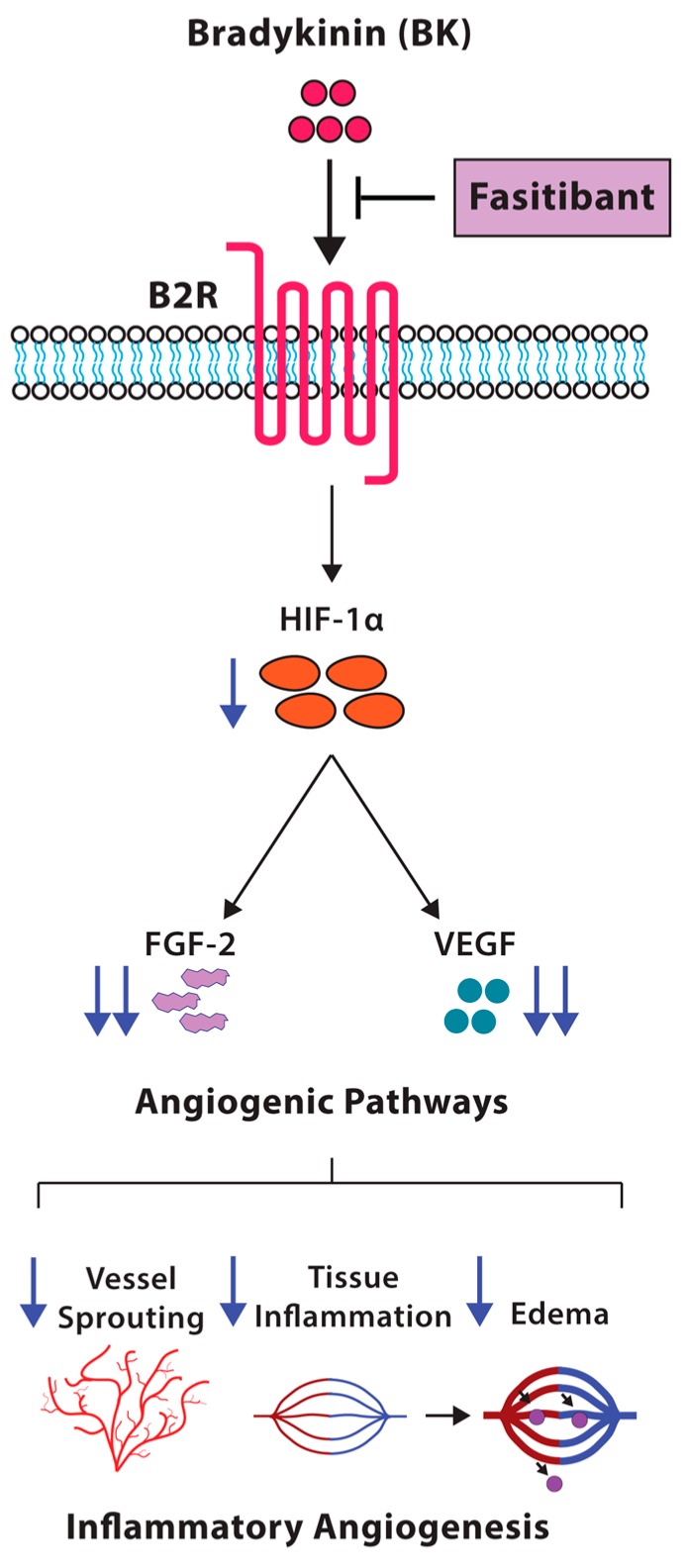
Schematic representation of the effects of inhibition of BK/B2R signaling on retinal vascularization. Fasitibant inhibits proangiogenic growth factor expression (FGF-2, VEGF, HIF-1α), reducing the inflammatory angiogenesis induced by BK/B2R signaling.

In this study, we demonstrated that the BK/B2R signaling affects the pattern of physiological retinal vascularization in mouse pups, and induces pathological inflammation-related neoangiogenesis in the model of rabbit cornea. In mice from OIR model, characterized by hypoxia-induced pathological retinal neovascularization, the blockade of B2R by the antagonist fasitibant significantly reduced retinal neovessels and expression of the proangiogenic and pro-inflammatory growth factors FGF-2 and VEGF (Figure 6).

The kallikrein–kinin system (KKS) has been implicated in the pathogenesis of retinal vascular inflammation and neoangiogenesis observed in several retinal diseases [2]. BK and its receptors B1R and B2R are the primary effectors of the KKS. B2R has been shown to mediate the pro-inflammatory and pro-angiogenic effects promoted by BK, which are central to the pathogenesis of diabetic retinopathy and macular edema [3]. Retinal inflammation plays a key role in the development of diabetic retinopathy, characterized by vessel permeability and upregulation of VEGF, nuclear factor-κB, cyclooxygenase 2, and prostaglandin-E2. We have demonstrated a similar pattern of inflammatory and proangiogenic molecules induced by BK/B2R system in endothelial cells and in a model of osteoarthritis [4]. Here, we demonstrate that B2R modulates physiological retinal vascularization, affecting the extent/sprouting, density and anastomosis of vessels. In a model of angiogenesis in the adult, the rabbit cornea assay, we show that activation of the B2R signaling is associated with corneal vessel sprouting and corneal opacification, strengthening the pathological relevance of these findings. Indeed, preservation of the avascular phenotype of the cornea has been associated with high levels of antiangiogenic factors as the soluble vascular endothelial growth factor receptor (sVEGFR1), able to neutralize the VEGF-A present in the cornea [22]. Thus, vascularization occurring during different pathophysiological conditions is the result of the perturbed balance among redundant inhibitory mechanisms. The recruitment of inflammatory cells by B2R further contributes to tissue detrimental reactivity. Consistently with these observations, the blockade of B2R in the OIR model significantly reduces the pathological pre-retinal neo-vascular tufts.

The half-life of BK in plasma is short [23], suggesting that its actions are regulated locally, and are propagated through stimulation of other local signaling. [24]. A limited number of studies suggests that the angiogenic response induced by BK/B2R system could be mediated by the upregulation of proangiogenic factors [25]. Although evidence for the direct activation of the FGF-2 or VEGF pathway by BK in vivo is not yet available, there is indirect evidence suggesting a functional link between the two systems in several inflammatory diseases, including vasoactive responses [17,18,19,20,26]. Notably, in this study, in the model of OIR, we observed that endothelial cells from retinal vessels express FGF-2 and VEGF, and that fasitibant reduced the expression of both growth factors, also marginally affecting HIF-1α expression. These data indicate that retinal endothelium is a target of the BK/B2R system and that B2R appears to modulate FGF-2 and VEGF signaling in retina vessels.

In light of the results, we propose a model for the angiogenic switch in retinal endothelial cells based on the BK/B2R signaling activation and, in turn, proangiogenic factor upregulation. We previously reported that FGFR-1 acts as a master switch in neovascularization mediated by inflammatory mediators by initiating a positive autocrine/paracrine cycle of FGF-2 synthesis and FGFR-1 activation [27]. Further studies are required to investigate whether BK promotes FGFR-1 signaling in retinal vessels. However, the downregulation of FGF-2 that we observed in the OIR model in response to B2R blockade suggests that the growth factor might be an important player in BK pro-inflammatory and proangiogenic effects. Similarly, BK proangiogenic activity has been reported to be mediated by the activation of VEGFR2 signaling [9]. Here, we demonstrated the involvement of the selective B2R signaling on VEGF expression, highlighting a role of the KKS in perpetuating signals for pathological retinal neovascularization triggers in ischemic proliferative disorders of the retina.

In conclusion, the blockade of the B2R signaling by a selective antagonist might restrict the pathological angiogenesis in retinal diseases, reducing the acute inflammatory and angiogenic responses of the vascular endothelium, and the concomitant amplification and propagation through the FGF-2 and VEGF pathway (Figure 6).

## 4. Materials and Methods

### 4.1. Fasitibant Treatment of Mouse Pups

Mice were housed in a controlled environment and provided with standard rodent chow and water. All animals were subjected to a 12 h light–12 h dark schedule. The experimental procedures, according to Italian (DL 26/2014) and European (No. 63/2010/UE) regulations on the protection of animals used for experimental and other scientific purposes, were approved by the Italian Ministry of Health (authorization No. 55/2017-PR, 20 January 2017). To investigate the role of B2R signaling on retinal vascularization, neonatal mice with their nursing mothers (C57Bl/6) were treated from postnatal day P3 until P8 (10/12 mice/experimental condition) daily with 0.5, 1 or 2 mg/kg fasitibant or NaCl 0.9% (control condition), in 50 µL, i.p. Mice were sacrificed at day 8 by cervical dislocation, and the eyes were enucleated for retinal dissection and evaluation of retinal vascularization by immunofluorescence and Western blot analysis.

### 4.2. Immunofluorescence Analysis

Enucleated eyes were immersion-fixed for 2 h in 4% paraformaldehyde in 0.1 M phosphate buffer (PB) at room temperature. Retinal dissection was performed as reported [28]. To visualize blood vessels, retinal whole mounts were incubated for 18 h at 4 °C with the endothelial isolectin, IB4-488 (Vector Laboratories, Cambridgeshire, UK) monoclonal antibody (1:50) diluted in 0.5% Triton X-100-containing 0.1 M PBS, and then incubated for 48 h at 4 °C in Alexa Fluor 488 (1:200) in 0.1 M PB. Finally, they were rinsed in 0.1 M PB, mounted on gelatin-coated glass slides, and cover-slipped with a 0.1 M PB-glycerine mixture. The antibody specificity was evaluated by the use of rabbit IgG negative control (1 μg/mL). Immuno-fluorescent materials were observed with confocal microscopy (Leica TCS SP5 confocal, Leica microsystems, Wetzlar, Germany. using ×10 or ×40 objective lens). Electronic images from the confocal microscope were processed using Adobe Photoshop 8.0 (Adobe Systems Incorporated, San Jose, CA, USA) [29].

### 4.3. Angiogenesis In Vivo: Rabbit Cornea Assay

The experimental procedures, according to Italian (DL 26/2014) and European (No. 63/2010/UE) regulations on the protection of animals used for experimental and other scientific purposes, were approved by the Italian Ministry of Health (Authorization n. 148/2015-PR, 6 March 2015).

The angiogenic activity was assessed in vivo by using the avascular rabbit cornea assay [30,31]. New Zealand white rabbits (Charles River, Calco, Como, Italy) (approx. 2 kg) were anesthetized by i.m. injection of xilazine 2% (0.5 mL/animal) and tiletamine/zolazepam (10 mg/kg). In the lower half of the eye, a micro pocket (1.5 × 3 mm) was surgically produced, and slow-release pellets (1 × 1 × 0.5 mm) with BK (1 μg, 3 eyes) or the selective B2R agonist kallidin (1 or 5 μg, 3 eyes for each condition) were implanted in the micropockets located inside the transparent corneal stroma. The corneas were observed every two other days for 2 weeks after implant, and digital images were taken by means of a slit-lamp stereomicroscope. Vessel sprouting was measured on digitalized images in a blind manner by the use of National Institute of Health (NIH)-ImageJ. Corneal opacity was also scored (from 0 to +++) at each observation.1.

### 4.4. The Mouse Model of OIR

Oxygen-induced retinopathy, OIR, is a reliable model of vascular retinopathy. In brief, neonatal mice with their nursing mothers (C57Bl/6) were exposed to 75% oxygen from postnatal day P7 until P12 and then returned to room air (21% oxygen) [32]. On return to room air, from P12 to P15 mice (10 mice/experimental condition) were treated daily with 2 mg/kg fasitibant or NaCl 0.9% (control condition), in 50 µL, i.p. At room air, the central vascular area becomes hypoxic, HIF-1α levels increase and promote the formation of pathologic neovessels (NV), also termed as pre-retinal tufts. Mice were sacrificed at day 16 by cervical dislocation, and the eyes were enucleated for retinal dissection and evaluation of retinal vascularization by immunofluorescence and Western blot analysis.

### 4.5. Western Blot Assay

Retinas (a pool of 4 retinas/experimental point) were homogenized and centrifuged at 22,000× *g* for 30 min at 4 °C. The pellet was re-suspended in 20 mM HEPES (4-(2-hydroxyethyl)-1-piperazineethanesulfonic acid), pH 7.4 containing 150 mM NaCl, 5 mM EDTA (Ethylenediaminetetraacetic acid), 3 mM EGTA (Ethylene-bis(oxyethylenenitrilo)tetraacetic acid), 1 mM phenylmethylsulphonyl fluoride, 1 μM peptistatin, 10 μg/mL leupeptin and 2 μg/mL aprotinin, and centrifuged at 22,000× g for 30 min at 4 °C. The supernatant was used to detect FGF-2, VEGF, HIF-1alpha and B2R. Proteins from cell extracts were electrophoresed in SDS (sodium dodecyl sulfate)/4–12% polyacrylamide gels (Life Technologies, Monza, MB, Italy). Proteins were then blotted onto activated nitrocellulose membranes, incubated overnight with the indicated antibodies, and antigen–antibody complexes were detected with enhanced chemiluminescence kit (Bio-Rad, Milan, Italy). Band intensity was measured by scanning densitometry.

### 4.6. Materials and Reagents

Cell culture reagents, BK, kallidin, anti-B2R and anti-β-actin were purchased from Sigma Aldrich (Merk Millipore, Darmstadt, Germany). Fasitibant was kindly provided by Menarini Ricerche, Florence, Italy. Anti-VEGF, anti-FGF-2 was from Merk Millipore (Darmstadt, Germany). Anti-HIF-1α was from Bioss (Aurogene, Rome, Italy).

### 4.7. Data Analysis and Statistical Procedures

Results are either representative or an average of at least three independent experiments done in triplicate. Statistical analysis was performed using a two-way ANOVA test followed by Bonferroni test (GraphPad Prism software, version 6.0c, La Jolla, CA USA). *p* < 0.05 was considered statistically significant.

## Figures and Tables

**Table 1:** Assessment of angiogenesis by BK and kallidin in the rabbit cornea assay.

Stimulus (Dose/Pellet)	Corneal Opacity (Day 2)	Sprouting (Day 8)	Sprouting (Day 14)
BK (1 μg)	++	3.6 ± 0.4	5.0 ± 0.1
Kallidin (1 μg)	++	1.8 ± 0.2	1.3 ± 0.9
Kallidin (5 μg)	+++	3.7 ± 0.9	4.6 ± 0.5

Corneal opacity was monitored and scored blind at every observation. The column reports data obtained at day 2. Numbers represent vessel sprouting (area in mm) from the limbus to the pellet implant during time from surgery. Data represents means ± standard deviation (SD) of three implants.

## Abbreviations

A.D.U.	Arbitrary density unit
BK	Bradykinin
B1R	BK receptor 1
B2R	BK receptor 2
Fas	Fasitibant
FGF-2	Fibroblast growth factor-2
HIF-1α	Hypoxia-inducible factor 1-alpha
HK	High–molecular-weight kininogen
i.p.	Intraperitoneal
KKS	Kallikrein–kinin system
NV	Neovessel
OIR	Oxygen-induced retinopathy
PB	Phosphate buffer
PBS	Phosphate buffer saline
VEGF	Vascular endothelial growth factor

## References

[1] Pruneau D., Bélichard P., Sahel J.A., Combal J.P. (2010). Targeting the kallikrein-kinin system as a new therapeutic approach to diabetic retinopathy. Curr. Opin. Investig. Drugs.

[2] Gao B.B., Clermont A., Rook S., Fonda S.J., Srinivasan V.J., Wojtkowski M., Fujimoto J.G., Avery R.L., Arrigg P.G., Bursell S.E. (2007). Extracellular carbonic anhydrase mediates hemorrhagic retinal and cerebral vascular permeability through prekallikrein activation. Nat. Med..

[3] Liu J., Feener E.P. (2013). Plasma kallikrein kinin system and diabetic retinopathy. Biol. Chem..

[4] Parenti A., Morbidelli L., Ledda F., Granger H.J., Ziche M. (2001). The bradykinin/B1 receptor promotes angiogenesis by up-regulation of endogenous FGF-2 in endothelium via the nitric oxide synthase pathway. FASEB J..

[5] Terzuoli E., Meini S., Cucchi P., Catalani C., Cialdai C., Maggi C.A., Giachetti A., Ziche M., Donnini S. (2014). Antagonism of bradykinin B2 receptor prevents inflammatory responses in human endothelial cells by quenching the NF-kB pathway activation. PLoS ONE.

[6] Bossi F., Peerschke E.I., Ghebrehiwet B., Tedesco F. (2011). Cross-talk between the complement and the kinin system in vascular permeability. Immunol. Lett..

[7] Hofman Z.L., Relan A., Zeerleder S., Drouet C., Zuraw B., Hack C.E. (2016). Angioedema attacks in patients with hereditary angioedema: Local manifestations of a systemic activation process. J. Allergy Clin. Immunol..

[8] D’Amore P.A. (1994). Mechanism of retinal and choroidal neovascularization. Investig. Ophthalmol. Vis. Sci..

[9] Thuringer D., Maulon L., Frelin C. (2002). Rapid transactivation of the vascular endothelial growth factor receptor KDR/Flk-1 by the bradykinin B2 receptor contributes to endothelial nitric-oxide synthase activation in cardiac capillary endothelial cells. J. Biol. Chem..

[10] Vinores S.A., Youssri A.I., Luna J.D., Chen Y.S., Bhargave S., Vinores M.A., Schoenfeld C.L., Peng B., Chan C.C., LaRochelle W. (1997). Upregulation of vascular endothelial growth factor in ischemic and non-ischemic human and experimental retinal disease. Histol. Histopathol..

[11] Monti M., Donnini S., Morbidelli L., Giachetti A., Mochly-Rosen D., Mignatti P., Ziche M. (2013). PKCε activation promotes FGF-2 exocytosis and induces endothelial cell proliferation and sprouting. J. Mol. Cell. Cardiol..

[12] Stavri G.T., Zachary I.C., Baskerville P.A., Martin J.F., Erusalimsky J.D. (1995). Basic fibroblast growth factor upregulates the expression of vascular endothelial growth factor in vascular smooth muscle cells. Synergistic interaction with hypoxia. Circulation.

[13] Yan Q., Li Y., Hendrickson A., Sage E.H. (2001). Regulation of retinal capillary cells by basic fibroblast growth factor, vascular endothelial growth factor, and hypoxia. In Vitro Cell. Dev. Biol. Anim..

[14] Calvani M., Rapisarda A., Uranchimeg B., Shoemaker R.H., Melillo G. (2006). Hypoxic induction of an HIF-1alpha-dependent bFGF autocrine loop drives angiogenesis in human endothelial cells. Blood.

[15] Castellon R., Hamdi H.K., Sacerio I., Aoki A.M., Kenney M.C., Ljubimov A.V. (2002). Effects of angiogenic growth factor combinations on retinal endothelial cells. Exp. Eye Res..

[16] Holborn M., Jahn K., Limb G.A., Kohen L., Wiedemann P., Bringmann A. (2004). Characterization of the basic fibroblast growth factor-evoked proliferation of the human Müller cell line, MIO-M1. Graefes Arch. Clin. Exp. Ophthalmol..

[17] Kaneko H., Terasaki H. (2017). Biological involvement in microRNA in proliferative vitreoretinopathy. Transl. Vis. Sci. Technol..

[18] Zittermann S.I., Issekutz A.C. (2006). Basic Fibroblast Growth Factor (bFGF, FGF-2) potentiates leukocyte recruitment to inflammation by enhancing endothelia adhesion molecule expression. Am. J. Path.

[19] Meini S., Maggi C.A. (2008). Knee osteoarthritis: A role for bradykinin?. Inflamm. Res..

[20] Xin L., Ellman B.M., Kroin J.S., Chen D., Yan D., Mikecz K., Ranjan K.C., Xiao G., Stein G.S., Kim S. (2012). Species-specific biological effects of FGF-2 in articular cartilage: Implication for distinct roles within the FGF receptor family. J. Cell. Biochem..

[21] Zubakova R., Gille A., Faussner A., Hilgenfeldt U. (2008). Ca^2+^ signalling of kinins in cells expressing rat, mouse and human B1/B2-receptor. Int. Immunopharmacol..

[22] Ambati B.K., Nozaki M., Singh N., Takeda A., Jani P.D., Suthar T., Albuquerque R.J., Richter E., Sakurai E., Newcomb M.T. (2006). Corneal avascularity is due to soluble VEGF receptor-1. Nature.

[23] Bader M. (2008). Cardiovascular Hormone Systems: From Molecular Mechanisms to Novel Therapeutics.

[24] Renné T., Schuh K., Müller-Esterl W. (2005). Local bradykinin formation is controlled by glycosaminoglycans. J. Immunol..

[25] Da Costa P.L.N., Sirois P., Tannock I.F., Chammas R. (2014). The role of kinin receptors in cancer and therapeutic opportunities. Cancer Lett..

[26] Morbidelli L., Parenti A., Giovannelli L., Granger H.J., Ledda F., Ziche M. (1998). B1 receptor involvement in the effect of bradykinin on venular endothelial cell proliferation and potentiation of FGF-2 effects. Br. J. Pharmacol..

[27] Finetti F., Donnini S., Giachetti A., Morbidelli L., Ziche M. (2009). Prostaglandin E(2) primes the angiogenic switch via a synergic interaction with the fibroblast growth factor-2 pathway. Circ. Res..

[28] Pitulescu M.E., Schmidt I., Benedito R., Adams R.H. (2010). Inducible gene targeting in the neonatal vasculature and analysis of retinal angiogenesis in mice. Nat. Protoc..

[29] Connor K.M., Krah N.M., Dennison R.J., Aderman C.M., Chen J., Guerin K.I., Sapieha P., Stahl A., Willett K.L., Smith L.E. (2009). Quantification of oxygen-induced retinopathy in the mouse: A model of vessel loss, vessel regrowth and pathological angiogenesis. Nat. Protoc..

[30] Ziche M., Morbidelli L., Masini E., Amerini S., Granger H.J., Maggi C.A., Geppetti P., Ledda L. (1994). Nitric oxide mediates angiogenesis in vivo and endothelial cell growth and migration in vitro promoted by substance P. J. Clin. Investig..

[31] Ziche M., Morbidelli L., Choudhuri R., Zhang H.-T., Donnini S., Granger H.J., Bicknell R. (1997). Nitric oxide-synthase lies downstream of vascular endothelial growth factor but not basic fibroblast growth factor induced angiogenesis. J. Clin. Investig..

[32] Stahl A., Connor K.M., Sapieha P., Chen J., Dennison R.J., Krah N.M., Seaward M.R., Willett K.L., Aderman C.M., Guerin K.I. (2010). The mouse retina as an angiogenesis model. Investig. Ophthalmol. Vis. Sci..

